# Validation to Spanish of nursing assessment scale for early diagnosis of delirium - Nu-DESC*

**DOI:** 10.17533/udea.iee.v41n2e03

**Published:** 2023-08-18

**Authors:** Ángela María Henao-Castaño, Linamaría Lozano González, Luz Omaira Gómez Tovar

**Affiliations:** 1 RN, PhD. Proffesor Universidad Nacional de Colombia. Email: angmhenaocas@unal.edu.co https://orcid.org/0000-0003-4203-0016 Universidad Nacional de Colombia Universidad Nacional de Colombia Colombia angmhenaocas@unal.edu.co; 2 RN. Hospital Universitario Nacional, Bogotá (Colombia). Email: lina.lozano@hun.edu.co https://orcid.org/0000-0002-3227-9152 Hospital Universitario Nacional Bogotá Colombia lina.lozano@hun.edu.co; 3 RN, PhD. Proffesor Universidad Surcolombiana, Neiva (Colombia). Email: logomezt@unal.edu.co https://orcid.org/0000-0003-1054-8697 Universidad Surcolombiana Universidad Surcolombiana Neiva Colombia logomezt@unal.edu.co

**Keywords:** delirium, intensive care units, validation study, psychometrics, delirio, unidades de cuidados intensivos, estudio de validación, psicometría, delírio, unidades de terapia intensiva, estudo de validação, psicometria

## Abstract

**Objective:**

This work aimed to determine the validity and reliability of the Colombian Spanish version of the Nursing Delirium Screening Scale (Nu-DESC).

**Methods:**

A psychometric study was conducted to achieve the goal of this study, which measured face validity, content validity, sensitivity, specificity and predictive values of the Nu-DESC.

**Results:**

Face validity obtained a total Aiken V of 0.89, and content validity showed a modified Lawshe index of 0.92. When Nu-DESC was applied to 210 adult patients hospitalized in the Intensive Care Unit, it was found that 14.2% had suspected delirium. The instrument showed a sensitivity of 91.6%, specificity of 95.6%, positive predictive value of 73.3%, negative predictive value of 98.8%, good internal consistency with Cronbach's α of 0.8 and good concordance according to Cohen's Kappa index of 0.788.

**Conclusion:**

The Spanish version of the Nu-DESC scale for Colombia has appropriate psychometric values for assessing delirium risk. In addition, this scale is easy to apply, so the adaptation of nursing personnel for its employability favors routine monitoring and timely detection of delirium.

## Resumen

**Objetivo:**

Determinar la validez y confiabilidad en su versión al español de Colombia de la escala de detección de delirium por enfermería (Nu-DESC, por sus siglas en inglés Nursing Delirium Screening Scale).

**Métodos:**

Estudio psicométrico, el cual midió la validez facial, de contenido, sensibilidad, especificidad y valores predictivos de la escala Nu-DESC.

**Resultados:**

La validez facial obtuvo un V de Aiken total de 0.89 y la validez de contenido mostró un índice Lawshe modificado de 0.92. Al aplicar Nu-DESC en 210 pacientes adultos hospitalizados en Unidad de Cuidados Intensivos se halló que 14.2% tuvo sospecha de delirium. El instrumento mostró una sensibilidad del 91.6%, especificidad de 95.6%, valor predictivo positivo de 73.3%, valor predictivo negativo de 98.8%, buena consistencia interna con α de Cronbach de 0.8 y buena concordancia según índice de Kappa de Cohen de 0.788.

**Conclusión:**

La versión en español para Colombia de la escala Nu-DESC tiene adecuados valores psicométricos para la valoración del riesgo de delirium. Además, esta escala es de fácil aplicación por lo que la adaptación del personal de enfermería para su empleabilidad favorece una rutinaria monitorización y detección oportuna del delirium.

## Resumo

**Objetivo:**

Determinar a validade e a confiabilidade da versão colombiana em espanhol da escala de detecção de delirium em enfermagem (Nu-DESC, por sua sigla em inglês Nursing Delirium Screening Scale).

**Métodos:**

Estudo psicométrico, que mediu a validade facial, validade de conteúdo, sensibilidade, especificidade e valores preditivos da escala Nu -DESC.

**Resultados:**

A validade facial obteve um V de Aiken total de 0.89 e a validade de conteúdo mostrou um índice de Lawshe modificado de 0.92. Ao aplicar o Nu-DESC em 210 pacientes adultos internados na Unidade de Terapia Intensiva, constatou-se que 14.2% apresentavam suspeita de delirium. O instrumento apresentou sensibilidade de 91.6%, especificidade de 95.6%, valor preditivo positivo de 73.3%, valor preditivo negativo de 98.8%, boa consistência interna com α de Cronbach de 0.8 e boa concordância segundo o índice Kappa de Cohen de 0.788.

**Conclusão:**

A versão em espanhol para a Colômbia da escala Nu-DESC possui valores psicométricos adequados para avaliação do risco de delirium. Além disso, essa escala é de fácil aplicação, de modo que a adaptação do pessoal de enfermagem para sua empregabilidade favorece o acompanhamento rotineiro e a detecção oportuna do delirium.

## Introduction

Delirium is a neurocognitive disorder that frequently occurs in critically ill patients, characterized by disorientation, memory impairment, psychomotor agitation, confusion and hallucinations;^1^ therefore, it is a symptom of brain damage.^2^ Its consequences are highly deleterious in patients since it causes more days of mechanical ventilation, longer stay in Intensive Care Units (ICU), higher risk of infections and even higher mortality.^3^^,^^4^ Thus, it is important to prevent delirium, starting with its timely detection,^5^ since this is one of the main measures for its prevention and treatment.^6^^,^^7^ The Pain, Agitation and Delirium (PAD) guidelines,^8^ the ABCDEF bundle^9^ and the Humanizing Intensive Care Units (HU-CI) project^10^ confirm that the assessment of delirium using validated scales should be performed frequently so that timely preventive measures can be developed, even from the suspicion or probability of its presence. 

There are validated tools for delirium diagnosis, like Confusion Assessment Methods in Intensive Care Units (CAM-ICU), and Intensive Care Delirium Screening Checklist (ICDSC). Further, the Prediction of Delirium in ICU patients scale (PRE-DELIRIC) assesses delirium risk in patients hospitalized in ICU. Although the literature presents these tools, not always are not apply due to some ICU boundaries, such as unknowledge of these,^11^ delirium naturalization, high workload, mistrust about the results of these tools in sedated, depressed or uncooperative patients,^12^ and by believing about their application can disrupt to the patients and their families.^13^ The boundaries to using delirium scales reduce nurses' opportunity to detect delirium.

However, the nurse role has a high relevance to measure, prevent and treat patients with delirium because nurses are leadership in the ICU, its participation in decision-making, its permanent communication with patients, family and the health team^14^ and its constant presence with the patient that facilitates early identification of the signs and symptoms of delirium. Thus, nurses must continue leading the timely detection of delirium, using other tools such as the *Nursing Delirium Screening Scale* (Nu-DESC), ^15^ with which most of the barriers that limit its detection can be overcome.

The Nu-DESC scale determines the suspicion of delirium. It has five dimensions, assessment, disorientation, inappropriate behavior, inappropriate communication, delusions/hallucinations, and psychomotor retardation, consistent with the criteria of the diagnostic manual of mental disorders.^1^ Thus, this tool is useful for evaluating patients with or without mechanical ventilation and with or without sedation. It was developed by Gaudreau *et al*.^15^ in 2005 with adequate reliability values, a sensitivity of 85.7% and a specificity of 86.8%, with only two cases of false positives (FP) and three false negatives (FN). In addition, it is very quick to apply because its administration can take less than two minutes.^16^ Based on these considerations, and taking into account the realities in some ICUs in Colombia where there is a high ratio of patients per nurse (six or more patients per nurse),^15^ the routine application of Nu-DESC is considered pertinent to achieve this, it is necessary to have the validation of this scale in Spanish. Thus, this study aimed to determine the validity and reliability of the Spanish version of the Nu-DESC scale in Colombia.

## Methods

### Type of study

A psychometric, exploratory-observational study was developed, which included the translation into Spanish and measurement of face validity, content validity, sensitivity, specificity, reliability and predictive values of the Nu-DESC scale developed by Gaudreau *et al*.^15^

### Populations

(i) For the translation process of the scale, a total of two translators participated, of which one was a specialist in medical translation and interpretation, and two were certified in Colombia; (ii) Five experts participated in the face, and content validation process, nurses and nurses specialized in critical care, with more than five years of experience in ICU, were knowledgeable in the management and prevention of delirium, since they had at least one publication related to the subject, and agreed to participate freely and voluntarily in the study; and (iii) To test the translated and validated version of Nu-DESC, it was applied in a multipurpose ICU that treats patients with all types of pathologies - cardiovascular, trauma, surgical, among others - of a fourth level university hospital. Patients 18 years of age and older hospitalized in the ICU and with a consciousness score according to the Richmond Agitation and Sedation Scale (RASS) between -3 and +3 were included. Those with any cognitive, psychiatric or neurological disorder reported in the clinical history or confirmed by relatives were excluded. Thus, 210 patients were included to whom the nurse applied the Nu-DESC scale, filling out the items based on the observation of the patient's behavior during the shift. In patients with orotracheal intubation, the nurse used nonverbal communication to verify orientation, communication and allusions.

### Translation

The Nu-DESC scale was translated from English to Spanish, with its cross-cultural adaptation and back-translation from Spanish to English, following the first six steps recommended by the International Society for Pharmacoeconomics and Outcome Research (ISPOR) guidelines:^17^

(i) Preparation. The authors of the original version were asked for their authorization and participation in the process, and they gave their approval and agreed to participate, thus providing explanations of the Nu-DESC dimensions; (ii) Direct translation. A translator specialized in medical translation, and a non-specialist translator were asked to translate the scale from English to Spanish independently. The two translators were given conceptual information on delirium and specifically on the content of the scale; (iii) Reconciliation between the two independent translations. A 98% concordance was found since, out of 474 words in the specialist translator's version, 465 words coincided with the non-specialist translator's version. Thus, a third translator and proofreader reconciled the nine words for which there was no concordance; (iv) Retranslation. An official translator retranslated the reconciled Spanish version into English; (v) Revision of the retranslation. This process was carried out by the work team that prepared the original English version of the scale; and (vi) Harmonization. A meeting was held with all the translators who participated and the researchers, where the final versions in Spanish and the retranslated version were reviewed, and conceptual aspects were analyzed, leading to the approval of the final version. The retranslated version was returned to the original authors, who approved it without requesting clarifications or modifications. The remaining steps (cognitive report, review of the results of the cognitive report, proofreading and final report) were carried out following the steps of the face and content validity.

### Facial validity

We continued with the facial validity process of the Nu-DESC scale as proposed by Sánchez & Echeverry,^18^ taking the assessment of the group of experts exclusively. ICU specialist nurses were considered experts for this study since they are the ones who should apply this scale, and these same experts participated in the content validation process; therefore, their inclusion and exclusion criteria are described in that section. Each expert assessed the clarity, coherence, relevance and sufficiency of the scale through Aiken's V method^19^ with a minimum acceptable score of 0.826, evaluated on a scale of 1 to 4, with 1 not meeting the criterion, 2 low level, 3 moderate level and 4 high level. Additional space was allowed for observations.

### Content validity

Content validity was assessed by categorizing each of the dimensions into three items: essential, useful but not essential, and nonessential. In addition, a space was left for observations in each of the dimensions. The modified Lawshe model was used,^20^ whose guidelines indicate that for a judgment of five experts, the minimum accepted value is 0.6. The methodological process of the expert judgment in the face and content validation took into account the following recommendations proposed by Escobar and Cuervo:^21^ (i) It was defined that the objective of the expert judgment was to perform the content validation of the Nu-DESC scale translated into Spanish; (ii) Selection of the judges. In this step, the criteria of Skjong and Wentwortht were used.^22^ The experts were considered to have experience in evidence-based decision-making or expertise, as evidenced by their studies, research, publications, position, experience, recognition, reputation in the community, availability and motivation to participate. Thus, we included nurses who were specialists in intensive care, of legal age, with at least five years of experience in critical care and who worked in third and fourth-level care institutions. Those whose experience in the ICU was exclusively in administrative activities were excluded; (iii) Explanation of the dimensions and indicators measured by each of the items of the instrument. This allowed the expert to evaluate the relevance of the item; (iv) Description of the objective of the instrument. The objective of Nu-DESC was included in each evaluation form so that the expert was contextualized, an aspect that increases the level of specificity of the evaluation; (v) Design of worksheets. They were designed according to the objectives of the evaluation; (vi) Inter-judge agreement was calculated based on Lawshe's modified content validity model; and (vii) Preparation of the trial conclusions, which are presented in the results.

### Criterion validity

Following the recommendations of Sánchez and Echeverry,^18^ the criterion validity process was carried out using the Confusion Assessment Method for Intensive Care Unit (CAM-ICU) instrument as the gold standard since this instrument has been validated in Colombia since 2010^23^ and is the most widely used for the diagnosis of delirium, and is recommended by the Latin-American and Iberian Guide for the delirium management.^24^

### Instruments

Two scales were used: (i) CAM-ICU: this instrument has four criteria, acute change or fluctuating course of mental status, inattention, altered level of consciousness and cognitive alterations. If the first and third criteria are altered, and the patient fails in two or more items of the second criterion, the patient is considered positive for delirium. If only the first criterion is altered and fails in two or more of the second, the fourth criterion is evaluated, and if it fails in more than one point of this, it is considered positive for delirium. Its Spanish version in Colombia has a K index of 0.79, a sensitivity of 79.4%, a specificity of 97.9%, a positive predictive value of 93.1% and a negative predictive value of 93%;^23^ and (ii) Nu-DESC (Nursing Screening Delirium Scale) was designed and validated in 2005 by Gaudreau *et al*,^15^ who estimated a sensitivity of 85.7% and a specificity of 86.8%. This scale contains 5 dimensions of rapid completion: disorientation, inappropriate behavior, inappropriate communication, delusions/hallucinations and psychomotor retardation. Each of these is scored 0 if absent, 1 if occasional and 2 if frequent. If the patient obtains a total score greater than or equal to 2, delirium is suspected, as specified by the creators of the original version of the scale.

### Collection of information after facial and content validation

The data measurement process was carried out through observation and was performed by two nurses specialized in intensive care, who were trained in the application of the two scales. Nurse M applied the CAM-ICU on the participants, and fifteen minutes later, nurse P applied the Nu-DESC scale on the same patients who were included in the study according to the criteria previously described in the participant's section. Each recorded their results in an independent database since both nurses were unaware of the results of the other scale, and the demographic and clinical information of the patients, taken from the medical records, was recorded in the same database. Subsequently, the two databases were pooled, compared to verify the information and unified into a single database containing all the results.

### Data analysis, reliability and internal and external validity of the scale

(i) For face validity, the Simple Concordance Index was used to find the degree of agreement among the evaluators, which reflects the number of agreements as a function of the total number of coding. The Aiken V was also used to establish face validity and the modified Lawshe model for content validity to analyze the study population. Descriptive statistics were used with measures of central tendency and dispersion for quantitative variables and relative and absolute frequencies for categorical variables; (ii) In the reliability analysis of the scale, Cronbach's alpha was calculated, and its internal and external validity with sensitivity, specificity, positive predictive values (PPV), negative predictive values (NPV), receiver operating characteristic curve (ROC) and Cohen's Kappa or concordance index, taking the cut-off point of the Nu-DESC scale > 2 following the specifications of the original version of the scale. The CAM-ICU and SPSS version 29 software were used as gold instruments for data analysis.

### Ethical considerations

The international ethical guidelines outlined in the Declaration of Helsinki and the Belmont Report, and those of Colombia according to Resolution 8430 of 1993 and Law 911 of 2004 were followed. Thus, this inquiry safeguarded the principles of justice because all patients had equal opportunity to participate, beneficence because this scale benefited the participants by giving them a better chance of detecting delirium and society in general since the possibility to use in the Colombian population. Furthermore, this study preserved autonomy because all participants (patients and experts who supported the translation and face and content validation) were free to enter and leave the study when they wished, expressed in the consent or informed waiver signature. 

The researcher asked patients to sign a consent form to use their health status information and to be assessed by the nurses who applied the instruments. The participation of the actors consisted of the translators contributing their knowledge with the translation of the scale, the experts contributing with the revision of the scale, and the researcher applying the scale to the patients. The data were treated according to the requirements of Law 1581 of 2012 and Decree 1377 of 2013 on data protection. This study was approved by the ethics committee of the institution where the study was applied, Hospital Universitario Nacional, by act No. 201909 of 2020.

## Results

The methodology section describes the translation process; It follows the ISPOR guide recommendations. Thus, the preparation and direct translation steps were carried out completely without novelties, the reconciliation step had a concordance between translators of 98%, an official translator performed the retranslation, the revision of the retranslation was performed directly by the creators of the original scale who did not suggest changes, and the final harmonization step involved the researchers, translators and creators of the original version, resulting in the final approval of the Spanish version published in this article. Subsequently, the facial and content validation process continued.

### Face Validity

Five critical care specialist nurses with more than five years of experience in ICU care (experts) participated in the study. They independently assessed the Spanish version of the Nu-DESC, from which this inquiry found that the clarity of all items (disorientation, inappropriate behavior, inappropriate communication, delusions/hallucinations and psychomotor retardation) had an Aiken V between 0.86 and 1, that is, between acceptable and perfect agreement among the experts. The coherence and relevance of the items were rated between 0.93 and 1 Aiken V, i.e. between high and perfect agreement, and the sufficiency was 0.93 for all items, i.e. high agreement (see Table 1). According to the observations of the experts, the word "evidence" was changed to "evidence for item 1, in item 2 an evaluator recommended adjusting the wording as follows: "Inappropriate behavior in space and/or time manifested by: throwing the pipe, pulling clothes, attempts to get off the bed." Another evaluator recommended adding inappropriate behaviors that would allow assessment of hypoactive delirium. On item 3 it was suggested to adjust the wording in the explanation as follows: "slow reaction to a stimulus or no spontaneous action to stimulus, delayed responses, patients evidenced as not resisting". Finally, regarding item 4, the experts considered it appropriate to exemplify illusions and hallucinations.

### Content Validity by experts

We determined content validity through an agreement among five experts with the degree of the agreement through the modified Lawshe model. We evaluated consensus among the experts with a content validity rank CVR (Content Validity Ratio) of at least 0.6. Table 1 presents the consolidated Aiken and Lawshe results for the Nu-DESC dimensions.

**Table 1:** Results of the Aiken and Lawshe V tests of Nu-DESC in Spanish, evaluation by five experts.

Ítem	Categories for determining face validity (V of Aiken)				Categories for determining content validity (Lawshe)		
	Clarity	Consistency	Relevance	Sufficiency	Essential	Useful but not essential	Not essential
Disorientation	1	1	1	0.93	1		
Inappropriate behavior	0.93	0.93	1	0.93	1		
Inappropriate communication	0.93	1	1	0.93	1		
Illusions/Hallucinations	1	1	1	0.93	1		
Psychomotor retardation	0.86	0.93	0.93	0.93		0.6	

The results showed that the content validity index for the Nu-DESC scale was 0.92. For the dimensions of disorientation, inappropriate behavior, inadequate communication and delusions/hallucinations had a Lawshe of 1, while for the psychomotor retardation item had a Lawshe of 0.6. Since an expert opinion generated a lower Lawshe in the psychomotor retardation category, it was not modified. These values corroborate adequate content validity. The validated Nu-DESC scale in its Spanish version is included in the Annex to this article.

### Characteristics of the patient population

We applied the scale to 210 adults hospitalized in the ICU of a university hospital in Bogotá. Data were collected between July and December 2021. The patients were mostly men (59.5%), mean age 60±15.2 years, with a predominance of diagnoses of cardiovascular pathologies (75.2%), sepsis (7.2%) and trauma (6.2%), and pathologic antecedents such as arterial hypertension (51.2%) and diabetes mellitus (27.7%). The pharmacological treatment of sedation and analgesia received by the participants was evaluated, with morphine (21.1%) being the most frequent. Other conditions found were the presence of mechanical ventilation in 19.7% and central venous catheter (34.7%) and urinary catheter (34.3%).

### Results of Nu-DESC and gold standard CAM-ICU

According to the CAM-ICU scale, 24 patients (11.4%) presented delirium, versus 30 patients (14.2%), according to Nu-DESC. Table 2 shows the results of each dimension, where disorientation and psychomotor retardation were the most frequent, and delusions/hallucinations were less frequent.

**Table 2:** Results of each Nu-DESC dimension in the study population

Dimension	Absent (0)	Ocassional (1)	Frequent (2)
	***n* (%)**	***n* (%)**	***n* (%)**
Disorientation	166 (77.9)	38 (17.8)	9 (4.2)
Inappropriate behavior	190 (89.2)	21 (9.9)	2 (0.9)
Inappropriate communication	180 (84.5)	30 (14.1)	3 (1.4)
Illusions/Hallucinations	201 (94.4)	10 (4.7)	2 (0.9)
Psychomotor retardation	174 (81.7)	36 (16.9)	3 (1.4)

### Reliability, internal and external validity results

**Figure1. ROC:**
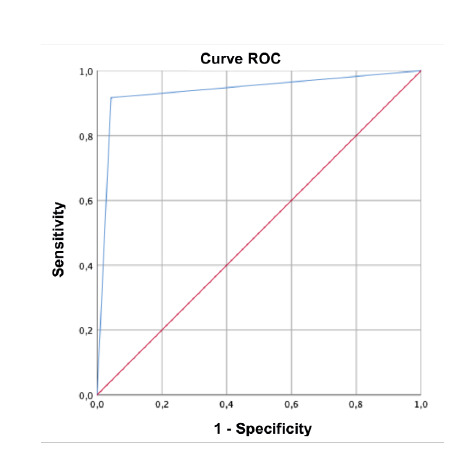
curve for the Spanish version of the Nu-DESC scale with a score > 2 points Versus CAM-ICU.

A Cronbach's alpha of 0.8 was estimated, indicating that the Nu-DESC scale's Spanish version is reliable. The ROC curve showed high sensitivity and specificity results according to the Youden index, closer to 1 in the upper left corner (Figure 1).

The area under the curve was 0.937±0.034 (p<0.001, 95%CI= 0.87-1), indicating the good ability of Nu-DESC to distinguish between positives and negatives.

In addition, the sensitivity showed that its ability to detect delirium in people with positive CAM-ICU was 91.6%, and the specificity of the scale showed that its ability to classify correctly without disease was 95.6%. The PPV estimated a 73.3% probability that an ICU patient with Nu-DESC score > 2 in Spanish has delirium. In comparison, NPV was a 98.8% probability of not having delirium when the scale score was < 2.

Cohen's Kappa index of the total Nu-DESC scale was 0.788 (p<0.001), i.e. good agreement. The results of Cohen's Kappa for each dimension show that the assessment of disorientation, inappropriate behavior and psychomotor retardation showed moderate agreement, inappropriate communication with good agreement, and delusions and/or hallucinations with poor agreement (Table 3).

**Table 3:** Cohen's Kappa index results for each dimension of the Spanish version of the Nu-DESC scale.

Dimension	Kappa	*p value*
Disorientation	0.472	<0.001
Inappropriate behavior	0.401	<0.001
Inappropriate communication	0.643	<0.001
Illusions/Hallucinations	0.382	<0.001
Psychomotor retardation	0.531	<0.001

## Discussion

The results of this study highlighted the validity of the Spanish version of the Nu-DESC scale. Its face validity showed a total Aiken V of 0.89, and its content validity showed a modified Lawshe index of 0.92. Likewise, Nu-DESC in Spanish has adequate internal and external validity since it had 91.6% sensitivity, 95.6% specificity, 73.3% PPV, and 98.8% NPV. Furthermore, we found Cronbach's α of 0.8 representing good internal consistency in the Spanish version of Nu-DESC, and Cohen's Kappa index of 0.788 indicates good agreement between Nu-DESC in Spanish and CAM-ICU.

We found few differences when comparing the present results with others reported in the literature. In 2017, Spedale *et al*.,^25^ published the Italian version of Nu-DESC, who obtained results similar to the present ones, with Kappa index of 0.87 and sensitivity of 100%, values slightly higher than the present ones in their Spanish version, but with a specificity of 76% lower than the present one, and the area under the ROC curve was 0.94, similar to the present one. Thus, the results of both versions are similar (Italian and Spanish), corroborating the overall reliability of the scale.

Moreover, in Denmark, Hägi-Pedersen *et al*.^26^ published the Danish version of Nu-DESC from its translation process that followed the ten steps of the ISPOR guide. In contrast, in the present one, the first six were followed. Like the Danish version, the current Spanish version relied on a team of translators and the original author of the scale to establish the final version. Similarly, the face and content validity varied in the number of participants since in the current version; there were five nurses and in the Danish version, 16 nurses and four physicians. There were also differences in the assessment items since the Danish version^26^ assessed comprehension and feasibility, and the current Spanish version set clarity, coherence, relevance and sufficiency. Finally, although there was variation in the final validation process, both versions conclude that this scale allows the timely detection of delirium.

We found that the results of the reliability tests of the present Spanish version of Nu-DESC were slightly superior to the German and Polish versions since, Brich *et al*.^27^ published the German version tested in 315 older adult participants, finding moderate sensitivity (66%), high specificity (91%), PPV of 7.37 and NPV of 0.37. Regarding the Polish version performed by Krupa *et al*.^28^ in 2021, their translation process was similar to the current one, obtaining a version adapted to their language and nation. Subsequently, the authors analyzed each of the dimensions and the global scale during the day and night and in two days of follow-up, finding that disorientation was the dimension with the highest frequency, a result similar to that of the present study.

Likewise, a study in Iran by Amirajam *et al*.^29^ performed psychometric tests of Nu-DESC English version in non-intubated ICU patients and found a Kappa of 0.96 and Cronbach's alpha of 0.86, which denote very good concordance and reliability. These results are higher than the present ones, although the differences are not significant, which may be due to the smaller population included in Amirajam's study (96 participants); however, Amirajam's inquiry confirms the efficiency and reliability of the scale.

Compared to the Thai version, translated and validated by Somnuke *et al*.^30^ in 2022 in a postoperative population over 70 years of age, it was observed that its sensitivity was low (55%) with a threshold ≥ 2. However, the sensitivity improved (85%) with a threshold of ≥ 1. The findings of Somnuke *et al*.^30^ are similar to those reported by Hargrave *et al*.^31^ about better internal validity with a threshold of 1; in addition, both authors had a population outside the ICU, which suggests that this scale may have variations in its cut-off point when assessing delirium in adults outside the ICU. Another study with the current Spanish version is needed to corroborate whether the same behavior is followed.

From another perspective, when analyzing the current Nu-DESC results against other instruments, such as the Recognizing acute delirium as part of your routine (RADAR) scale,^32^ it has three simple items based on observation, drowsiness, difficulty following instructions, and slow movements. Nurses applied RADAR every time they administered medication, as they wanted it to be part of the care routine. The results of its validation showed a sensitivity of 73% and specificity of 67%, which are lower than those of the validation of the first English version of Nu-DESC and the current ones.

Another instrument developed was a computerized device called the Edinburgh Delirium Test Box-ICU, which detected and monitored visual deficits and delirium.^33^ It consisted of a behavioral assessment and a computerized test, with which patients had to slowly count the lights presented to them. Khan *et al*.^34^ compared Test Box-ICU and CAM-ICU results in 30 ICU patients, and the authors found that their scores < 5 achieved 100% sensitivity and 92% specificity for detecting delirium. These figures were partially similar to those of the present study and corroborate the usefulness of other tools for assessing delirium, in the case of Test Box-ICU using modern technology that supports patient valuation.

On the other hand, when applying the Nu-DESC, a prevalence of 14.2% of patients with suspected delirium was found, similar to that found by Brich *et al*.^27^ in Germany, who found 14.9% in a population of 315 patients. At the same time, in the study by Krupa *et al*.^28^ in Poland, they detected between 24.3% and 67.3% of suspected delirium during two days of follow-up in a population of 202 participants. This result confirms delirium symptoms in adults and the need for measures to guide its prevention or treatment.

One limitation of this study was that the acceptance of the nursing staff for its application was not evaluated, which may be useful in future studies. Likewise, other validations of this version can be carried out in contexts other than the ICU, such as hospitalization or geriatrics, since delirium is also frequent in these services.

The results allow us to conclude that the Spanish version of the Nu-DESC scale for Colombia has adequate psychometric values for assessing the risk of delirium; in addition, it is a scale that is easy and quick to apply. Therefore, the Spanish version of Nu-DESC is valid and reliable for detecting suspected delirium in adult ICU patients. Suppose the scale is equal to or higher than 2. In that case, it should be reported to the physician to confirm the diagnosis and take the respective preventive or early treatment measures, as indicated in the clinical practice guidelines for managing pain, anxiety and delirium. Thus, it is recommended that the nurse should apply it to ICU patients on each shift.

## Appendix. Validated Spanish version of the Delirium Detection Scale for Nurses (Nu-DESC)

Instructions: For each shift, score each of the four behaviors as follows: 0 = Behavior that did not occur during the shift; 1 = Behavior that occurred at some point during the shift but slightly, occasional or mild; 2 = Behavior that occurred at some point during the shift and was marked, frequent or intense. In the end, add the total, and if a total score < 1 is interpreted as NO SUSPECT OF DELIRIUM and a result ≥ 2 is considered SUSPECT OF DELIRIUM, in this case, please apply the method for the assessment of confusion in intensive care (CAM-ICU) to confirm the diagnosis.

**Table 4:** Validated Spanish version of the Delirium Detection Scale for Nurses (Nu-DESC)

	Turno / Shift		
Fecha (día / mes / año):	Mañana	Tarde	Noche
Date (day / month / year):	Mornign	Afternoon	Evening
1. Desorientación: manifestación verbal o comportamental de una pérdida de orientación temporal o espacial, o una percepción equivocada de las personas en el entorno			
Disorientation: Verbal or behavioral manifestation of not being oriented to time or place or misperceiving persons in the environment.			
2. Comportamiento inapropiado: Comportamiento inapropiado para el lugar y/o la persona; por ejemplo, halar de mangueras o vendajes, intentar levantarse de la cama cuando está contraindicado y comportamientos por el estilo			
Inappropriate behavior: Behavior inappropriate to place and/or for the person; e.g., pulling at tubes or dressings, attempting to get out of bed when that is contraindicated, and the like.			
3. Comunicación inapropiada: Comunicación inapropiada para el lugar y/o la persona; por ejemplo, incoherencia, falta de comunicación, forma de hablar que no tiene sentido o no puede entenderse			
Inappropriate communication: Communication inappropriate to place and/or for the person; e.g., incoherence, noncommunicativeness, nonsensical or unintelligible speech.			
4. Ilusiones o alucinaciones: Ver o escuchar cosas que no están ahí, distorsiones de objetos visuales			
Illusions/Hallucinations: Seeing or hearings things that are not there; distortions of visual objects.			
5. Retraso psicomotor: respuesta retardada, pocas o ningunas acciones o palabras espontáneas			
Psychomotor retardation: delayed responsiveness, few or no spontaneous actions/words.			
Puntaje total / Total score			

## References

[1] American Psychiatric Association (2013). Diagnostic and Statistical Manual of Mental Disorders.

[2] Page VJ, Wesley E (2015). Delirium in Critical Care.

[3] Reznik ME, Slooter AJC (2019). Delirium Management in the ICU. Curr. Treat. Options Neurol.

[4] Tran NN, Hoang TPN, Ho TKT (2021). Diagnosis and risk factors for delirium in elderly patients in the emergency rooms and intensive care unit of the national geriatric hospital emergency department: A cross-sectional observational study. Int. J. Gen. Med..

[5] Gómez Tovar LO, Henao Castaño ÁM (2021). Interventions and effectiveness of the ABCDEF Package in the treatment of delirium: scoping review. Av. Enferm..

[6] Devlin JW, Skrobik Y, Gélinas C, Needham DM, Slooter AJC, Pandharipande PP (2018). Clinical Practice Guidelines for the Prevention and Management of Pain, Agitation/Sedation, Delirium, Immobility, and Sleep Disruption in Adult Patients in the ICU. Crit. Care Med..

[7] Balas MC, Weinhouse GL, Denehy L, Chanques G, Rochwerg B, Misak CJ (2018). Interpreting and Implementing the 2018 Pain, Agitation/Sedation, Delirium, Immobility, and Sleep Disruption Clinical Practice Guideline. Crit. Care Med..

[8] Garrett KM. (2016). Best Practices for Managing Pain, Sedation, and Delirium in the Mechanically Ventilated Patient Sedation Agitation Pain Delirium Mechanical ventilation. Crit. Care Nurs. Clin. North Am..

[9] Marra A, Wesley E, Pandharipande PP, Patel MB (2017). The ABCDEF Bundle in Critical Care. Crit. Care Clin..

[10] Velasco Bueno JM, Heras La Calle G, Ortega Guerrero Á, Gómez Tello V (2017). Manual of Good Practices of Humanization in Intensive Care Units.

[11] Gómez Tovar LO, Henao-Castaño M, Troche-Gutiérrez IY (2022). Preventing and treating delirium in intensive care: hermeneutics of the nursing team's experiences. Enferm. Intensiva..

[12] Zamoscik K, Godbold R, Freeman P (2017). Intensive care nurses’ experiences and perceptions of delirium and delirium care. Intensive Crit. Care..

[13] Oosterhouse KJ, Vincent C, Gruss VA, Corte C, Berger B (2016). Intensive Care Unit Nurses’ Beliefs About Delirium Assessment and Management. Adv. Crit. Care..

[14] Von Rueden KT, Wallizer B, Thurman P, McQuillan K, Andrews T, Merenda J (2017). Delirium in Trauma Patients: Prevalence and Predictors. Crit. Care Nurse..

[15] Gaudreau JD, Gagnon P, Harel F, Tremblay A, Roy MA (2005). Fast, systematic, and continuous delirium assessment in hospitalized patients: The nursing delirium screening scale. J. Pain Symptom Manage..

[16] van Velthuijsen EL, Zwakhalen SMG, Warnier RMJ, Mulder WJ, Verhey FRJ, Kempen GIJM (2016). Psychometric properties and feasibility of instruments for the detection of delirium in older hospitalized patients: a systematic review. Int. J. Geriatr. Psychiatry..

[17] Wild D, Grove A, Martin M, Eremenco S, McElroy S, Verjee-Lorenz A (2005). Principles of good practice for the translation and cultural adaptation process for patient-reported outcomes (PRO) measures: Report of the ISPOR Task Force for Translation and Cultural Adaptation. Value Heal..

[18] Sanchez R J E. (2004). Validacion de escalas de medicion en salud. Rev. Salud Pública..

[19] Escurra Mayaute LM. (1969). Cuantificación de la validez de contenido por criterio de jueces. Rev. Psicol..

[20] Tristán-López A. (2008). Modification of Lawshe's model for quantitative judgement of the content validity of an objective instrument. Av. Medición..

[21] Escobar-Pérez J, Cuervo-Martínez Á (2008). Content Validity and Expert Judgment: An Approach to Its Use. Av. Medición.

[22] Skjong R, Wentworth BH (2001). Expert judgment and risk perception. Proc. Int. Offshore Polar Eng. Conf..

[23] Toro AC, Escobar LM, Franco JG, Díaz-Gómez JL, Muñoz JF, Molina F (2010). Spanish version of the method for the evaluation of confusion in intensive care, pilot validation study. Med. Intensiva..

[24] Celis-Rodríguez E, Díaz Cortés JC, Cárdenas Bolívar YR, Carrizosa González JA, Pinilla DI, Ferrer Záccaro LE (2020). Evidence-based clinical practice guidelines for the management of sedoanalgesia and delirium in critically ill adult patients. Abstract.. Med. Intensiva..

[25] Spedale V, Di Mauro S, Del Giorno G, Barilaro M, Villa CE, Gaudreau JD (2017). Delirium assessment in hospitalized elderly patients: Italian translation and validation of the nursing delirium screening scale. Aging Clin. Exp. Res..

[26] Hägi-Pedersen D, Thybo KH, Holgersen TH, Jensen JJ, Gaudreau JD, Radtke FM (2017). Nu-DESC DK: The Danish version of the nursing delirium screening scale (nu-DESC). BMC Nurs..

[27] Brich J, Baten V, Wußmann J, Heupel-Reuter M, Perlov E, Klöppel S (2019). Detecting delirium in elderly medical emergency patients: validation and subsequent modification of the German Nursing Delirium Screening Scale. Intern. Emerg. Med..

[28] Krupa S, Dorota O, Friganovic A, Mędrzycka-Dąbrowska W, Jurek K (2021). The polish version of the nursing delirium screening scale (Nudesc pl)-experience of using in nursing practice in cardiac surgery intensive care unit. Int. J. Environ. Res. Public Health..

[29] Amirajam Z, Asadi-Noran E, Molaei B, Adiban V, Heidarzadeh M, Hassanpour-Darghah M (2021). Psychometric properties of nursing delirium screening scale in patients admitted to intensive care units. Indian J. Crit. Care Med..

[30] Somnuke P, Limprapassorn P, Srinonprasert V, Wongviriyawong T, Suraarunsumrit P, Morkphrom E (2022). The Thai version of the nursing delirium screening scale-Thai: Adaptation and validation study in postoperative patients. Front. Med..

[31] Hargrave A, Bastiaens J, Bourgeois JA, Neuhaus J, Josephson SA, Chinn J (2017). Validation of a Nurse-Based Delirium-Screening Tool for Hospitalized Patients. Psychosomatics.

[32] Voyer P, Champoux N, Desrosiers J, Landreville P, Mccusker J, Monette J (2015). Recognizing acute delirium as part of your routine [RADAR]: a validation study. BMC Nursing..

[33] Green C, Hendry K, Wilson ES, Walsh T, Allerhand M, MacLullich AMJ (2017). A Novel Computerized Test for Detecting and Monitoring Visual Attentional Deficits and Delirium in the ICU. Crit. Care Med..

[34] Khan BA, Perkins AJ, Gao S, Hui SL, Campbell NL, Farber MO (2017). The Confusion Assessment Method for the ICU-7 Delirium Severity Scale: A Novel Delirium Severity Instrument for Use in the ICU. Crit. Care Med..

